# Langerhans Cell Homeostasis and Activation Is Altered in Hyperplastic Human Papillomavirus Type 16 E7 Expressing Epidermis

**DOI:** 10.1371/journal.pone.0127155

**Published:** 2015-05-18

**Authors:** Nor Malia Abd Warif, Patrizia Stoitzner, Graham R. Leggatt, Stephen R. Mattarollo, Ian H. Frazer, Merilyn H. Hibma

**Affiliations:** 1 University of Queensland Diamantina Institute, Translational Research Institute, 37 Kent Street, Woolloongabba, Brisbane, Qld 4102, Australia; 2 Department of Dermatology and Venereology, Medical University of Innsbruck, Innsbruck, Austria; 3 Department of Pathology, Dunedin School of Medicine, University of Otago, Dunedin, New Zealand; Oklahoma Medical Research Foundation, UNITED STATES

## Abstract

It has previously been shown that expression of human papillomavirus type 16 (HPV) E7 in epidermis causes hyperplasia and chronic inflammation, characteristics of pre-malignant lesions. Importantly, E7-expressing epidermis is strongly immune suppressed and is not rejected when transplanted onto immune competent mice. Professional antigen presenting cells are considered essential for initiation of the adaptive immune response that results in graft rejection. Langerhans cells (LC) are the only antigen presenting cells located in normal epidermis and altered phenotype and function of these cells may contribute to the immune suppressive microenvironment. Here, we show that LC are atypically activated as a direct result of E7 expression in the epidermis, and independent of the presence of lymphocytes. The number of LC was significantly increased and the LC are functionally impaired, both in migration and in antigen uptake. However when the LC were extracted from K14E7 skin and matured *in vitro* they were functionally competent to present and cross-present antigen, and to activate T cells. The ability of the LC to present and cross-present antigen following maturation supports retention of full functional capacity when removed from the hyperplastic skin microenvironment. As such, opportunities are afforded for the development of therapies to restore normal LC function in hyperplastic skin.

## Introduction

Squamous cell carcinomas (SCC) result from transformation of the epidermal keratinocytes (KC). They arise from premalignant precursor lesions such as actinic keratoses or squamous cell carcinoma *in situ* [1], typically on UV exposed skin [2] or in the case of SCC of the cervix as a result of infection with high-risk human papillomavirus types. HPV type 16 E7 is a cell cycle deregulating protein that contributes to the oncogenesis of HPV16, a high-risk type that is primarily associated with cervical cancer. Expression of HPV16 E7 in the keratinocytes (KC) of the epidermis is sufficient to regulate KC cell growth and differentiation [3]. E7-expressing transgenic mouse skin (K14E7) displays the hallmark characteristics of premalignant skin (hyperplasia and hyperkeratosis), contains chronic inflammatory infiltrates that parallel those of epithelial cancers in humans [4], and SCC develops in these mice late in life [5]. Additionally, the skin of the K14E7 mouse is immune suppressed and is not rejected when transplanted onto immune competent mice that should recognise the E7 as a foreign antigen [6]. The K14E7 mouse therefore has utility in understanding regulation of immunity in hyperplastic, premalignant skin.

Langerhans cells (LC) are langerin (CD207) positive dendritic cells that form a network amongst the KC of the epidermis. They are professional antigen presenting cells (APC), constitutively expressing MHCII and CD11c. In their immature state, LC are competent to take up and process antigen [7]. Following migration from the epidermis, LC express high levels of MHCII and costimulatory markers and are potent stimulators of T cells *in vitro* [8]. A role for LC in T cell maintenance in the periphery is also now recognized, such that LC induce proliferation of skin resident T cells in an antigen specific manner [9].

LC are implicated in the immune regulation of T cells in the skin. LC contribute to maintenance of grafts, supporting T cell-mediated suppression [10]. In contrast, at least in the LC-depletable Langerin-DTR transgenic mouse, LC are mediators of chronic hypersensitivity (CHS) responses, and CHS is suppressed when LC are depleted [11]. The capacity of LC to regulate the expansion of effector and regulatory skin resident T cells is dependent on the skin microenvironment [9]. The effects of the microenvironment on LC in hyperplasia are largely unknown.

The purpose of this study is to establish if LC number and function is affected in the skin of the K14E7 mouse, to better understand how they are regulated and if they may contribute to regulation of immunity in premalignant skin. In this study we found that LC were increased in number and their migration was impaired in E7-expressing epidermis. The LC were activated and antigen uptake was impaired. However when matured *ex vivo*, LC from the K14E7 mouse epidermis were competent to present and cross-present antigen and to prime T cells. These cells therefore display an atypical, altered phenotype and function when resident in the K14E7 hyperplastic skin. That these cells are competent to present and cross-present following maturation *ex vivo* suggests signaling from the local microenvironment is required for maintenance of the altered phenotype in the K14E7 epidermis.

## Materials and Methods

### Animals

Female C57BL/6, K14E7 (H-2^b^), RagKO and RagOTI mice aged between 6 and 10 weeks were obtained from the Animal Resources Centre (Perth, Australia). OTII mice were obtained from the Walter and Eliza Hall Institute (Melbourne, Australia). K14E7.RagKO mice were generated by crossing male K14 mice with female RagKO mice. Mice were humanely euthanized by CO_2_ asphyxiation. Experiments were performed in compliance with the ethical guidelines of the National Health and Medical Research Council of Australia, with approval from the University of Queensland Animal Ethics Committee.

### Antibodies

The following antibodies to mouse antigens were used: phycoerythrin (PE) anti-CD45.2 (104; eBioscience), phycoerythrin-indotricarbocyanine (PE-Cy7) anti-CD11c (HL3; BD Bioscience); anti-MHC class II (I-A/I-E) (M5/114.15.2; eBioscience); anti mouse-CD86 (clone GL-1; Biolegend), anti-CD80 (16-10A1, Biolegend); FITC anti-CD40 (HM40-3; BD Bioscience) and Alexa-546 anti-CD207 (929F3.01, Dendritics). Isotype-matched control antibodies were obtained from BD Biosciences. Flow count fluorospheres (Beckman Coulter) were used to determine absolute cell counts.

### Cell isolation

Ears were separated with forceps into dorsal and ventral halves. Epidermal cells were isolated by trypsinisation of epidermal sheets following a protocol modified from Koch [12]. The separated ear skin was floated dermis-side down on 0.5% trypsin in PBS for 25–45 min. at 37°C. Epidermis was separated from the dermis and incubated for 30 min at 37°C in Iscove’s medium containing 10% heat-inactivated fetal calf serum (FCS). The cells in suspension were filtered through a 70 μm cell strainer and pelleted by centrifugation (400 x *g* for 4 minutes at 4°C).

For the preparation of lymph node cell suspensions, inguinal lymph nodes were removed and digested with collagenase D (Roche) for 25 min at 37°C in RPMI-1640 containing 2% heat inactivated-FCS, followed by incubation with 0.01M EDTA for 5 min. The digested cells were filtered through cell strainers and washed thoroughly in PBS-EDTA-FCS.

### Preparation of tissues for immunofluorescence staining

To prepare epidermal sheets ears were split and floated dermal side down on 0.5 M ammonium thiocyanate (Sigma-Aldrich, A7149) for 20 min at 37°C [13]. Epidermis was peeled from the dermal layer and fixed in acetone for 10 min at RT. To prepare frozen tissue, ear skin was embedded in OCT (Sakura Finetek), snap frozen at -20°C and sectioned at 5–8 μm thickness. Sections were fixed in acetone at RT for 10 min prior to staining.

### Antigen uptake assay

To measure antigen uptake, epidermal cell suspensions (3 x 10^6^ cells in 1ml) were incubated with OVA labeled with 0.1 mg/ml Alexa-555 (Invitrogen) overnight at 37°C. Cells were harvested, stained and uptake of OVA-555 by the CD45.2, MHCII, CD207+ cells was measured using flow cytometry. For confocal analysis CD207+/MHCII+ cells were sorted, fixed with 4% formalin and cytospun onto glass slides prior to confocal microscopy.

### Langerhans cell migration

To measure migration, mouse skin was painted on a 15 mm^2^ area of shaved abdomen with 5mg/ml FITC in acetone and dibutylphtalate (1:1) 48 h prior to harvest of the inguinal lymph nodes. Single cell suspensions were prepared and the number of FITC positive Langerhans cells was determined.

### *Ex vivo* T cell proliferation assay

To test if LC from K14E7 mice could be matured *ex vivo*, cells were cultured for 72 h in the presence of 2ng/ml GM-CSF-supplemented media. To test presentation and cross-presentation of OVA epidermal cell suspensions were incubated with OVA overnight, washed extensively, cultured for a further 48 h in 2ng/ml GM-CSF-supplemented media then purified to 70–80% purity using anti-MHCII magnetic cell separation. Purified cells were co-cultured with purified OT-I or OT-II cells at ratios ranging from 0:1 to 1000:1 for 60 h and T cell proliferation was measured by incorporation of ^3^H-thymidine during a further 16 h incubation.

To test the ability of LC to prime T cells, epidermal cell suspensions were incubated with GM-CSF for 72 h, purified, incubated with the MHCI-restricted OVA peptide SIINFEKL or the MHCII-restricted OVA peptide ISQAVHAAHAEINEAGR for 2 h, washed then co-cultured with purified OTI or OTII cells for 72 h. ^3^H-thymidine was added for an additional 16 h and incorporation measured.

### Flow cytometry

Cell suspensions were resuspended in PBS containing 1% FCS and 0.1% sodium azide and incubated for 15 min at RT with the appropriate antibodies or isotype controls. For CD207 staining, cells were fixed and permeabilised using the Cytofix/cytoperm (BD BioSciences) according to the manufacturer’s recommendations. Following treatment, cells were stained with anti-mouse CD207 for 30 min at RT. Flow cytometric analysis was performed using the FACSCanto (BD Biosciences).

### Immunofluorescence staining

Fixed epidermal sheets (placed on glass slides), or frozen sections, were washed once in PBS before treated with PBS containing 2.5% FCS (blocking buffer) for 15 min at RT. Tissue were subsequently incubated with anti-CD207 or an isotype-matched control for 1 h at 37°C. After washing, tissues were mounted with Slowfade Gold (Invitrogen) and images were acquired using an LSM510 confocal microscope (Zeiss).

### Statistical Analysis

Pair-wise comparisons were carried out using Mann-Whitney U test with Prism (GraphPad) software. Analysis of lymphoproliferation assays was carried out using 2-way analysis of variance (ANOVA). Error bars on graphs represent standard error of the mean. *P* > 0.05 is not significant (*ns*); *P < 0.05; ** *P* < 0.01, *** *P* < 0.001.

## Results

### Number and morphology of LC from normal and E7-expressing epidermis

**Fig 1:**
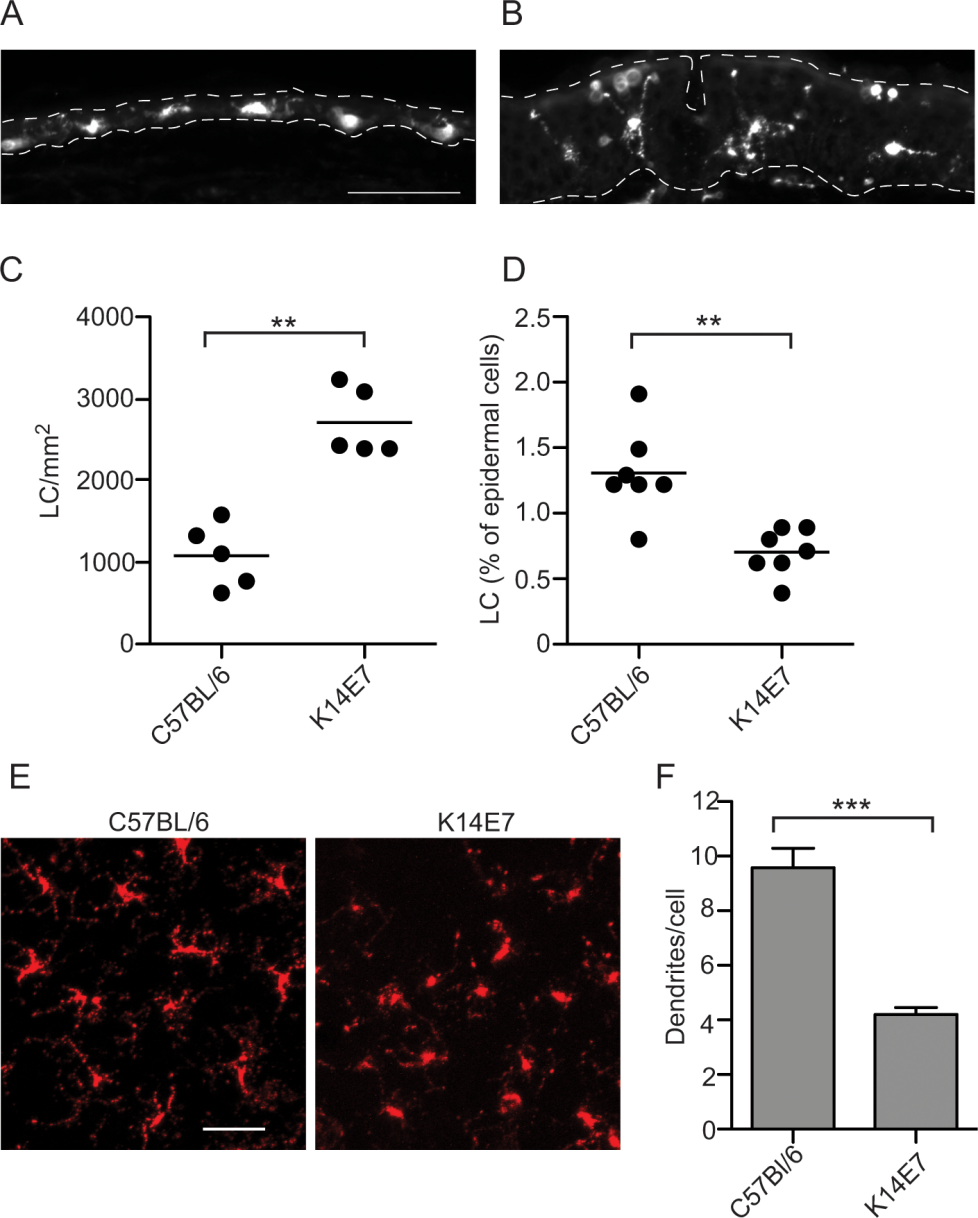
The absolute number of LC is increased in K14E7 mouse epidermis. Sections of C57Bl/6 (A) and K14E7 (B) mouse ear were stained with CD207 and imaged by epifluorescence microscopy. The epidermis (top) is indicated by a broad dashed line, and the epidermis/dermis interface by a narrow dashed line; scale bar = 50μm. LC (CD45.2, CD11c, CD207, MHCII+) harvested from a 5.5 mm diameter sample of dorsal ear epidermis were enumerated, with counting beads, by flow cytometry. The absolute number (C) and the percentage of LC (D) are shown. Confocal imaging was carried out on CD207-stained sheets of dorsal ear skin (E). Scale bar represents 20μm. Dendrites on the CD207+ LC were enumerated following confocal imaging (F). **P < 0.01 (Mann-Whitney U test).

It has previously been reported that more LC are present in high-grade cervical intraepithelial lesions, where expression of the HPV oncoproteins is typically increased [14, 15]. A thorough analysis of the number of LC in E7-expressing epidermis was carried out. Epithelium from K14E7 and control mice was sectioned then stained with the CD207/langerin-specific antibody. Compared with normal epidermis (Fig 1A), the epidermis of the K14E7 mouse displayed characteristic hyperplasia (Fig 1B), and the LC were located throughout the thickened epidermis. In order to determine if the number of LC in the epidermis was affected by E7 expression in basal keratinocytes, epidermal cells were isolated from K14E7 and C57Bl/6 mice and LC were enumerated using flow cytometry. The absolute number of LC harvested from K14E7 mouse ear skin was more than twice that of the equivalent C57BL/6 ear skin (Fig 1C). In contrast, the proportion of LC relative to KC was reduced in the K14E7 mouse skin when compared to control skin (Fig 1D), reflecting the increased number of KC as a consequence of the epidermal hyperproliferation caused by E7. Immunofluorescence staining with CD207 and confocal microscopy was carried out on epidermal sheets to identify LC and to visually validate the enumeration data. Consistent with the flow cytometry data, there was a greater density of LC in the epidermis from K14E7 mouse skin (Fig 1E), although overall the distribution was less uniform. The characteristic dendritic processes that are readily identifiable on the LC in the epidermis of the C57BL/6 mice were significantly less frequent on LC in K14E7 skin (Fig 1F). Taken together, the increased number of LC, the decreased ratio of LC to KC and their more rounded appearance, consistent with activated LC, support altered regulation of LC homeostasis and phenotype in K14E7 skin.

### Antigen uptake by LC from normal and K14E7 epidermis

**Fig 2:**
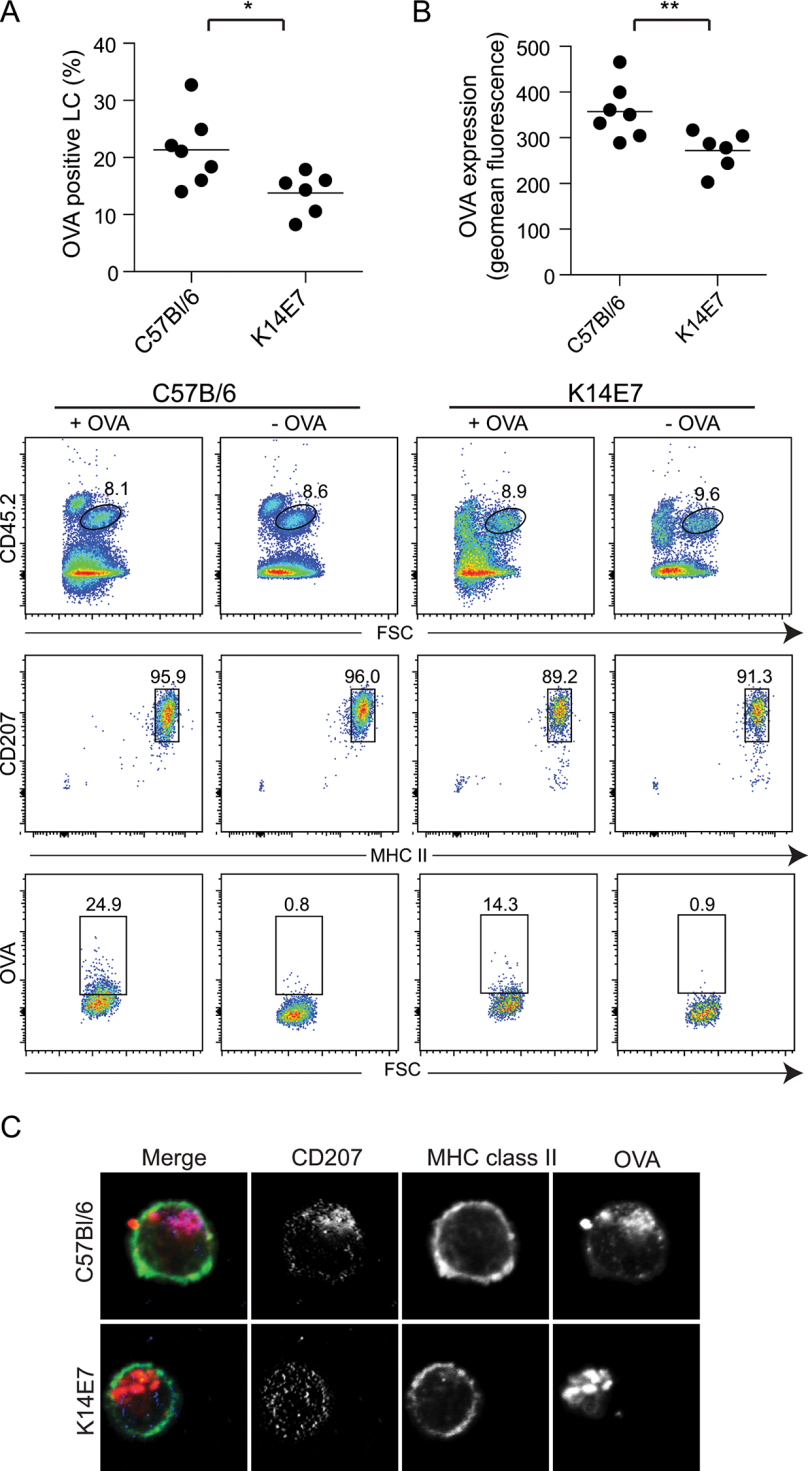
Antigen uptake is reduced in K14E7 mice. Epidermal cell suspensions from C57BL/6 and K14E7 mice were cultured with Alexa-555-labelled OVA overnight at 37°C, stained and analysed by flow cytometry (A). The Alexa-555-OVA+ LC (CD45.2+, MHC Class II+, CD207+) as a percentage of total LC (B) and the level of expression of Alexa-555-OVA on the positive stained LC is shown (C). **P < 0.01; *P < 0.05 (Mann-Whitney U test). Confocal imaging of CD207+, MHCII+ LC that have taken up Alexa-555-OVA (D).

We went on to examine if LC function was altered in K14E7 skin. To investigate whether endocytic uptake of a soluble protein antigen was affected in LC from K14E7 skin, epidermal cell suspensions from C57Bl/6 and K14E7 mouse skin were cultured overnight with fluorescently-labeled ovalbumin (OVA), harvested, immuno-labeled with CD45, MHCII and CD207 to identify the LC, and analysed by flow cytometry (Fig 2A). Around half of the LC from the K14E7 mouse epidermal cultures were OVA positive compared with the C57Bl/6 LC, and there was a modest but significant overall reduction of OVA fluorescence in the OVA positive LC from the K14E7 (Fig 2B and 2C). Using confocal microscopy it was confirmed that the OVA taken into the cell was cytoplasmic and was in a staining pattern consistent with localization to endosomes (Fig 2D). Interestingly, OVA appeared to be colocalised with langerin in LC from C57Bl/6 mice, whereas the distribution of langerin was punctate throughout the cell and did not co-localise in the LC from the K14E7 mice. Overall, these data show that when cultured with antigen fewer LC from the K14E7 skin took up the antigen, and those LC took up less antigen than LC from control skin.

### LC migration to the lymph node in the normal and K14E7 mouse

**Fig 3:**
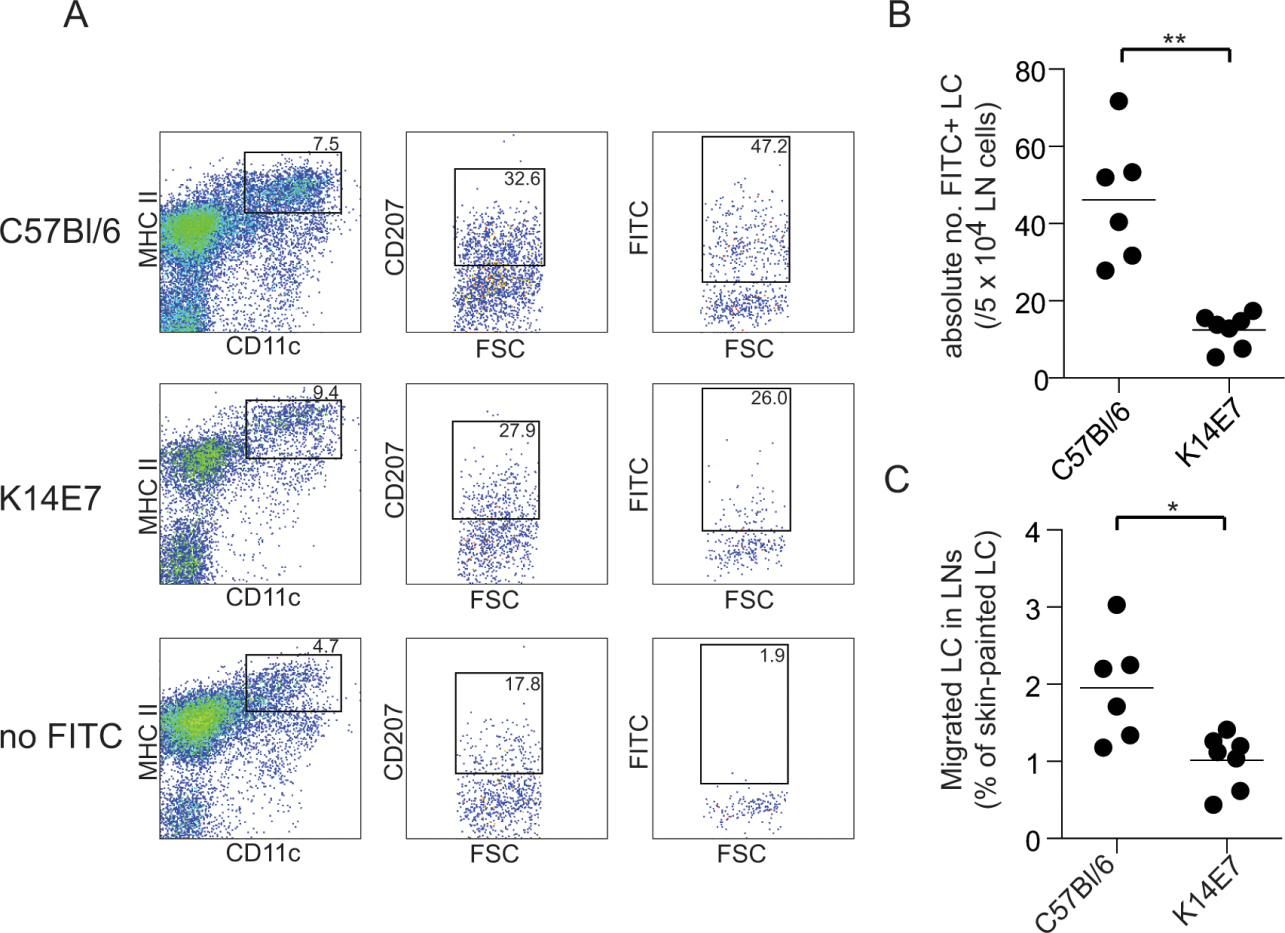
LC migration from the epidermis is reduced the in K14E7 mice. C57Bl/6 and K14E7 mice were painted with FITC on a 15mm^2^ area of abdomen and inguinal lymph nodes harvested 48 h later. Single cell suspensions were stained with CD11c, MHC class II and CD207 (A). The absolute number of FITC positive cells was determined (B). The total migrated LC in the lymph nodes as a percentage of the mean total number of LC in the painted skin is shown (C). *P < 0.05 (Mann-Whitney U test).

The increased numbers of LC in the K14E7 epidermis may be a result of an inability of the LC to migrate from the epidermis, leading to their accumulation. To assess the migratory ability of the K14E7 LC, mouse skin was painted with the contact sensitizer fluorescein isothiocyanate (FITC), and the absolute number of fluorescent migrating LC was counted. The percentage of LC that migrated to the LN in the K14E7 mouse 48 h after FITC painting was around half of that of the C57BL/6 control mice (Fig 3A and 3B). From this we conclude that migration of LC from the skin in K14E7 mice was impaired compared with control skin, at 48h post activation.

### Costimulatory molecule expression on LC from normal and K14E7 skin

**Fig 4:**
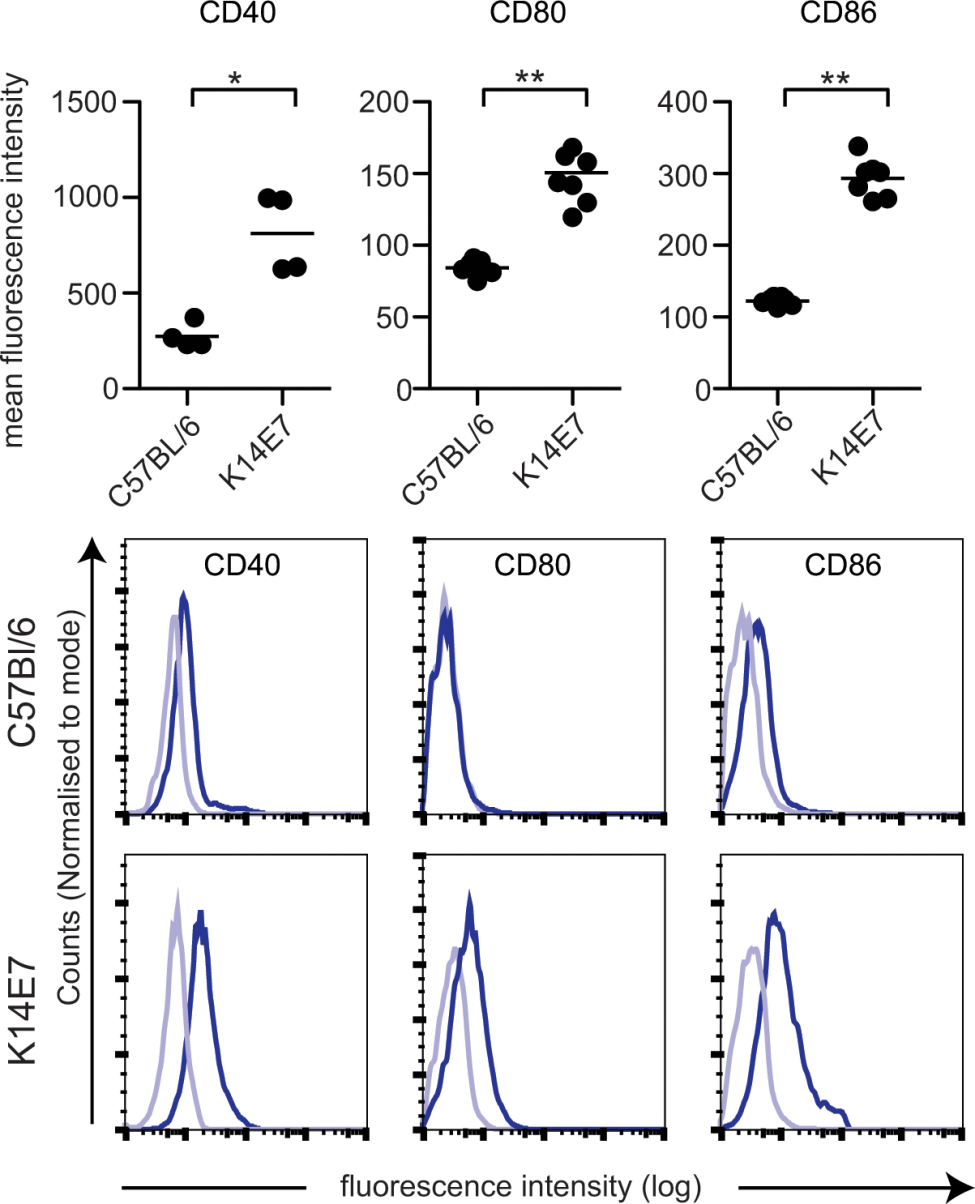
Co-stimulatory molecules are upregulated on epidermal LC from K14E7 mice. Epidermal suspensions were prepared from K14E7 and control mice and stained with antibodies to CD45.2, CD11c, CD207 to identify LC and CD40, CD80 and CD86 to determine their levels of expression. Expression of CD40, CD80 and CD86 (dark line) and isotype staining (light blue line) on LC is shown for representative flow cytometry data (A) and graphically for all data (B). ***P < 0.001; **P < 0.01; *P < 0.05 (Mann-Whitney U test).

T cell activation follows engagement of the TCR with MHC I or MHC II, and engagement of co-stimulation markers that include CD40, CD80 and CD86 on the antigen presenting cell. We questioned if expression of costimulatory molecules was altered on the LC from the K14E7 mouse epidermis. Epidermal cell suspensions were prepared from K14E7 and control mice and the expression of CD40, CD80 and CD86 on the LC was measured. Expression of each of the co-stimulatory markers tested here was somewhat increased on LC extracted from K14E7 mouse epidermis, compared to LC from control mice (Fig 4). Thus the LC from the K14E7 hyperplastic skin costimulatory molecule expression was consistent with partial activation.

**Fig 5:**
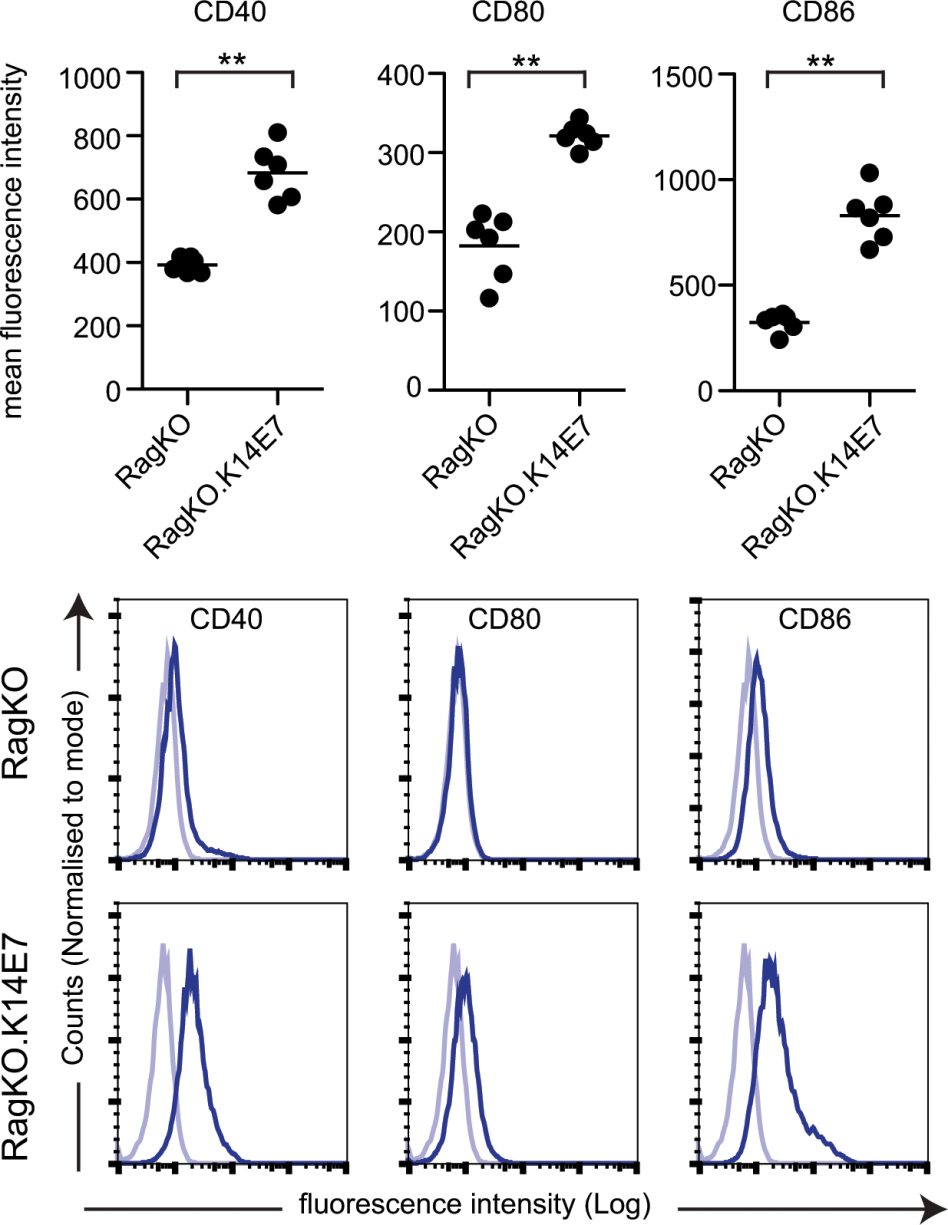
Increased expression of costimulatory molecules by E7 occurs in the absence of functional lymphocytes. Epidermal cell suspensions prepared from RagKO and K14.RagKO mice were stained with antibodies to CD45.2, CD11c, CD207 to identify LC and CD40, CD80 and CD86 to determine their levels of expression. Expression of CD40, CD80 and CD86 (dark line) and isotype staining (light blue line) on LC is shown for representative flow cytometry data (A) and graphically for all data (B). **P < 0.01 (Mann-Whitney U test).

Molecular crosstalk from T cells can alter the phenotype of dendritic cells, including co-stimulatory molecule expression [16]. To test the requirement for functional T lymphocytes in the increased surface molecule expression that we observed on LC from the K14E7 mouse skin, these mice were crossed with RagKO mice. The increased expression of CD40, CD80 and CD86 that we observed in the K14E7 mice was also detected in the K14E7xRagKO mice, in the absence of functional T cells (Fig 5). Therefore up-regulation of costimulatory molecules on epidermal LC from the K14E7 mouse is independent of the presence of functional lymphocytes in the skin.

### K14E7 LC capacity to prime T cells compared with LC from normal skin

**Fig 6:**
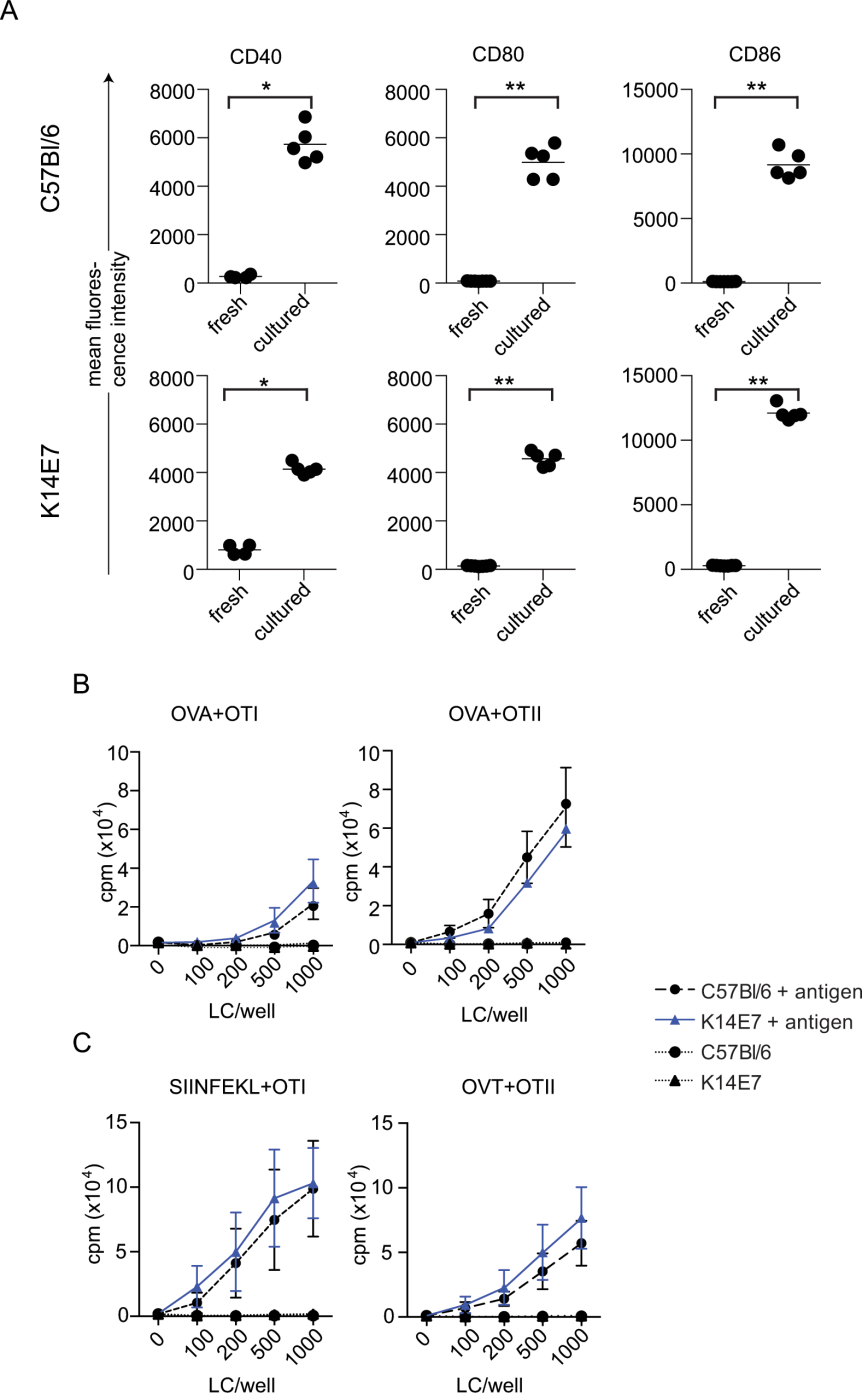
Co-stimulatory molecule expression is increased, and presentation and cross-presentation by LC from K14E7 mice does not differ from LC from C57Bl/6 mice. Expression of CD40, CD80 and CD86 was compared on CD45.2, CD11c, CD207 positive LC from C57Bl/6 and K14E7 mice prepared either directly from epidermal suspensions (fresh) or from epidermal cells that were matured in culture for 72 h in medium containing GM-CSF (A). Epidermal cells from C57BL/6 and K14E7 mice were incubated with OVA, cultured for 2 days then LC purified and co-cultured with purified OT-I or OT-II cells for 60 h. T cell proliferation was measured after addition ^3^H-thymidine for a further 16h (B). Epidermal cells were cultured for 72 h in GM-CSF, LC purified and pulsed with SIINFEKL or ISQAVHAAHAEINEAGR for 2 h, washed and co-cultured with T cells for 60 h. T cell proliferation was measured after addition ^3^H-thymidine for a further 16h (C). Data was analysed using 2-way ANOVA and there was no significant difference between the groups.

LC extracted from skin can be induced to mature *in vitro* in the presence of GM-CSF [17]. We found that LC from K14E7 mice expressed significantly higher levels of CD40, CD80 and CD86 on the cell surface following culture for 72 h in the presence of GM-CSF, compared with LC obtained directly from the epidermis and stained (Fig 6A). When compared with cultured control cells, there was some variability in levels of expression of each of the markers on K14E7 cells, with no significant difference (M-W) in CD80 expression, reduced CD40 expression (M-W; *P* < 0.01) and increased CD86 expression (M-W; *P* < 0.05). However overall, co-stimulatory marker expression was significantly increased on cells from either the K14E7 or the control mouse skin when compared to fresh cells, indicating cell maturation in culture had occurred.

The ability of LC from K14E7 skin to present and cross-present antigen to prime T cells was measured. Epidermal cell suspensions from K14E7 or control mice were pulsed with OVA and matured by culture in GM-CSF. The matured LC were purified, co-cultured with OT-I or OT-II cells, and T cell proliferation measured. There was no difference in the ability of matured LC from the K14E7 mouse to cross-present OVA to OTI cells or to present OVA to OTII cells (Fig 6B). The ability of the matured LC to prime T cells following incubation with SIINFEKL or ISQAVHAAHAEINEAGR was also tested. There was no difference in the ability of the matured LC to prime OT-I or OT-II cells *ex vivo* (Fig 6C).

## Discussion

Our data show that LC number and phenotype is altered in the hyperplastic, chronic inflammatory epidermis of the K14E7 mouse. Although several studies have reported that LC numbers are reduced in HPV16-infected low-grade cervical tissue [18], LC are increased in high-grade cervical intraepithelial neoplasia [14, 15]. Key differences in high-grade lesions that may contribute to regulation of LC number include increased expression of HPV oncoproteins and the resultant hyperproliferation and chronic inflammation. In support of this, increased LC are associated with chronic inflammatory conditions in humans, for example lichen planus [19], where they are proposed to have a role in the pathogenesis of the disease [20]. We propose that the secondary effects of E7 on the epidermis, including hyperplasia and chronic inflammation, contribute to the increased number of LC found there.

There was a pronounced difference in the morphology on the LC in the K14E7 mouse skin. Cells were more rounded and less dendritic, when compared with the control cells. Resting LC regularly extend and retract their dendrites between KC [21] however dendritic processes elongate when LC sense pro-inflammatory signals [22]. The retracted dendrites of LC from K14E7 epidermis may indicate differences in LC regulation between proinflammatory epidermis and the chronically inflamed, hyperproliferative K14E7 epidermis, where only selected proinflammatory cytokines are expressed [23]. Additionally, Cdc42 is a Rho-family GTPase that may be implicated in the LC phenotype we observe here. LC in Cdc42-deficient epidermis are less dendritic and display a rounded phenotype, consistent with what we observe here, and do not migrate to the lymph node following activation [24].

Although we have not established if the kinetics of LC migration was altered, we did find that emigration of LC from the K14E7 hyperplastic epidermis is reduced at 48h post activation, which may contribute to an accumulation of LC in the skin. This may seem surprising as expression of IL-1β, TNF-α and IL-18, cytokines associated with migration of LC from the skin, are all increased in K14E7 epidermis [25, 26]. However high levels of TNF-α inhibit LC migration [27] and IL-10, which is also inhibitory [28], is significantly increased in E7-expressing epidermis [26]. Regulation of cytokine expression in E7-expressing KC is therefore a likely contributor to the inhibition of LC emigration that we observed here.

The increase in LC may also result from an influx of LC precursors into the skin. During tissue trauma and acute inflammation, LC are repopulated by circulating Gr1^hi^ blood monocytes that differentiate into LC in the epidermis [29, 30]. LC immigration is mainly controlled by increased expression by KC of the chemoattractant MIP3α/CCL20 [31]. We see increased levels of CCL20 RNA (manuscript in preparation) in K14E7 mouse skin, suggesting that this may be the case.

Proliferation of LC resident in the epidermis is essential for LC homeostasis [32], but may also contribute to increased LC numbers in the hyperproliferative epidermis in the K14E7 mouse. Induction of atopic dermatitis in the mouse results in epidermal proliferation, causing skin thickening and inflammation and an increase in LC numbers. There was strong local proliferation of LC that was attributed to unidentified factor or factors produced by the hyperproliferative keratinocytes [33]. It is feasible that secretion of one or more LC proliferation-inducing factors produced by the E7-expressing, hyperplastic KC has a growth effect on LC in the local microenvironment that may also contribute to LC expansion in the K14E7 mouse epidermis.

Expression of CD40, CD80 and CD86 were increased on LC in the K14E7 epidermis. Expression of co-stimulatory molecules, including CD80 and CD86, is increased on mature LC, and normally occurs during migration of LC to the lymph nodes [34–36]. However increased CD80 and CD86 are observed in chronically inflamed skin conditions, such as atopic dermatitis [37]. Mechanistically, HPV16 E7 increases NFκB activity in KC to induce expression of selected proinflammatory cytokines [23], which may account for the increased CD80 and CD86 expression on the neighbouring LC. In addition, iNKT cells infiltrate K14E7 mouse skin [38]. These cells regulate IFNγ [38], which induces CD80 and CD86 expression on APC [39]. IFNγ produced by lymphocytes may contribute to increased CD80 and CD86 but does not appear to be of primary importance mechanistically as expression of these molecules was also increased on LC from K14E7xRag-/- mice, in the absence of functional lymphocytes.

The K14E7-derived LC displayed impaired antigen uptake that when cultured with OVA-555. OVA uptake is mediated by macropinocytosis [40] and clathrin-mediated endocytosis via C-type lectins [41]. Also langerin contains EPN-motif shared by the C-type lectins in the mouse DC-sign family and can mediate uptake of glycosylated proteins such as OVA [42]. The co-localisation of langerin with OVA in LC from the C57Bl/6 mice that we observed supports its involvement in the uptake of OVA. Interestingly, langerin association with OVA taken up by LC has been shown in immature LC, whereas although mature LC are readily able to take up OVA, it does not colocalise with langerin in the LC [43]. Consistent with this, although langerin expression was not altered in the K14E7 LC (data not shown), OVA and langerin were not co-localised. Sparber, et al. (2010) suggested that different uptake pathways were used at different stages of LC maturation. Indeed this may be the case, as the LC from the K14E7 skin expressed increased CD40, CD80 and CD86, consistent with a more mature state. Upregulation of CD40, CD80 and CD86 on LC is typical of a state of activation, for example following hapten painting [17, 44]. The increased expression of co-stimulatory markers indicates that cells are somewhat activated in the chronic inflammatory and hyperproliferative K14E7 epidermis.

The LC from C57Bl/6 skin were efficient cross-presenters of exogenous antigen, as has been reported previously [45]. We were surprised to see that LC extracted from K14E7 and control skin and matured following culture with GM-CSF did not differ in their capacity to present or cross-present antigen to T cells *in vitro*. Although the phenotype of the LC from the K14E7 mouse was substantially altered when resident in the skin, these cells retained the capacity to be further matured and to present and cross-present antigen. From this we conclude that the altered phenotype of the LC from the K14E7 mice is regulated by the skin microenvironment but is reversible when cells are removed from the skin and matured *ex vivo*.

Here we show that LC in hyperplastic E7-expressing skin are increased in frequency and are partially activated either as a direct consequence of E7 expression or as a result of the effects of E7 on the skin microenvironment. These cells are held in a state where if harvested and tested directly for antigen uptake, their ability to do so is impaired. Additionally, their migration to the lymph nodes is impaired *in vivo*. However when the LC are removed from the skin microenvironment and further differentiated in culture, their competence to present antigen and provide co-stimulatory signals to T cells is intact. This suggests that the functional regulation of LC from E7-expressing skin is local, dependent on the E7-skin microenvironment, but also may be reversible. We therefore speculate that the function of LC from E7 may be restored using immune modifying strategies, which could be applied for therapeutic benefit against HPV infection in the event that LC do contribute to the immune suppression seen in K14E7 mouse skin.
